# Multiple Primary Lung Cancers With ALK Rearrangement: A Case Report and Literature Review

**DOI:** 10.3389/fonc.2022.897451

**Published:** 2022-05-23

**Authors:** Zhou Huimin, Wang Xueting, Qi Qi, Feng Lingxin, Yang Xue, Yu Zhuang, Wang Jing

**Affiliations:** Department of Oncology, the Affiliated Hospital of Qingdao University, Qingdao, China

**Keywords:** multiple primary malignant neoplasms, small cell lung cancer, non-small cell lung cancer, combined small cell lung cancer, anaplastic lymphoma kinase

## Abstract

Multiple primary lung cancers (MPLCs) are that patients with lung cancer may present with two primary tumors at the same time (synchronous multiple primary lung cancer, SMPLC) or may develop a second, metachronous primary lung cancer after treatment of the initial lesion. Currently, there are no definitive guidelines for the diagnosis and treatment of multiple primary lung cancers. Herein, we report a case of double primary lung cancers with ALK rearrangement. The patient was treated with chemotherapy, targeted therapy, and radiotherapy. After these treatments, the patient was free of locally recurrent or distant disease at 2 years.

## Introduction

We report a case of double primary lung cancers (DPLCs) with ALK rearrangement and review the literature. The patient has provided her written informed consent for the publication of this manuscript and any identifying images or data. DPLC is one type of MPLC. MPLC is divided into synchronous MPLC (sMPLC) and metachronous MPLC (mMPLC). As the incidence of lung cancer soars, the diagnoses of patients with multiple primary lung cancers (MPLCs) increase (1, 2). Martini and Antakli et al. (3, 4) proposed the clinical and pathological diagnostic criteria of MPLCs. Furthermore, if MPLC is isolated and has no distant metastasis, surgical resection is still necessary (5). Therefore, invasive mediastinal staging and extrathoracic imaging (head computed tomography/magnetic resonance plus whole-body positron emission tomography or abdominal computed tomography plus bone scan) are recommended for patients with tumors located at different lung lobes.

Multiple primary lung cancers have many characteristics, one of which is that the focus has different histologic types or different molecular genetic characteristics or arises separately from foci of carcinoma *in situ* (3, 5), which provides us with a lot of help for the diagnosis of multiple primary lung cancers in the future. With the discovery of immunohistochemistry and genetic testing, the probability of multiple primary lung cancer detection increases gradually. In order to diagnose lung tumors, especially in cases with similar histopathological types, we can evaluate them by means of genotype or immunoassay, such as epidermal growth factor receptor (EGFR) and tumor protein 53 (TP53). It can provide not only evidence for diagnosis but also direction for follow-up treatment (6).

## Case Presentation

**Figure 1:**
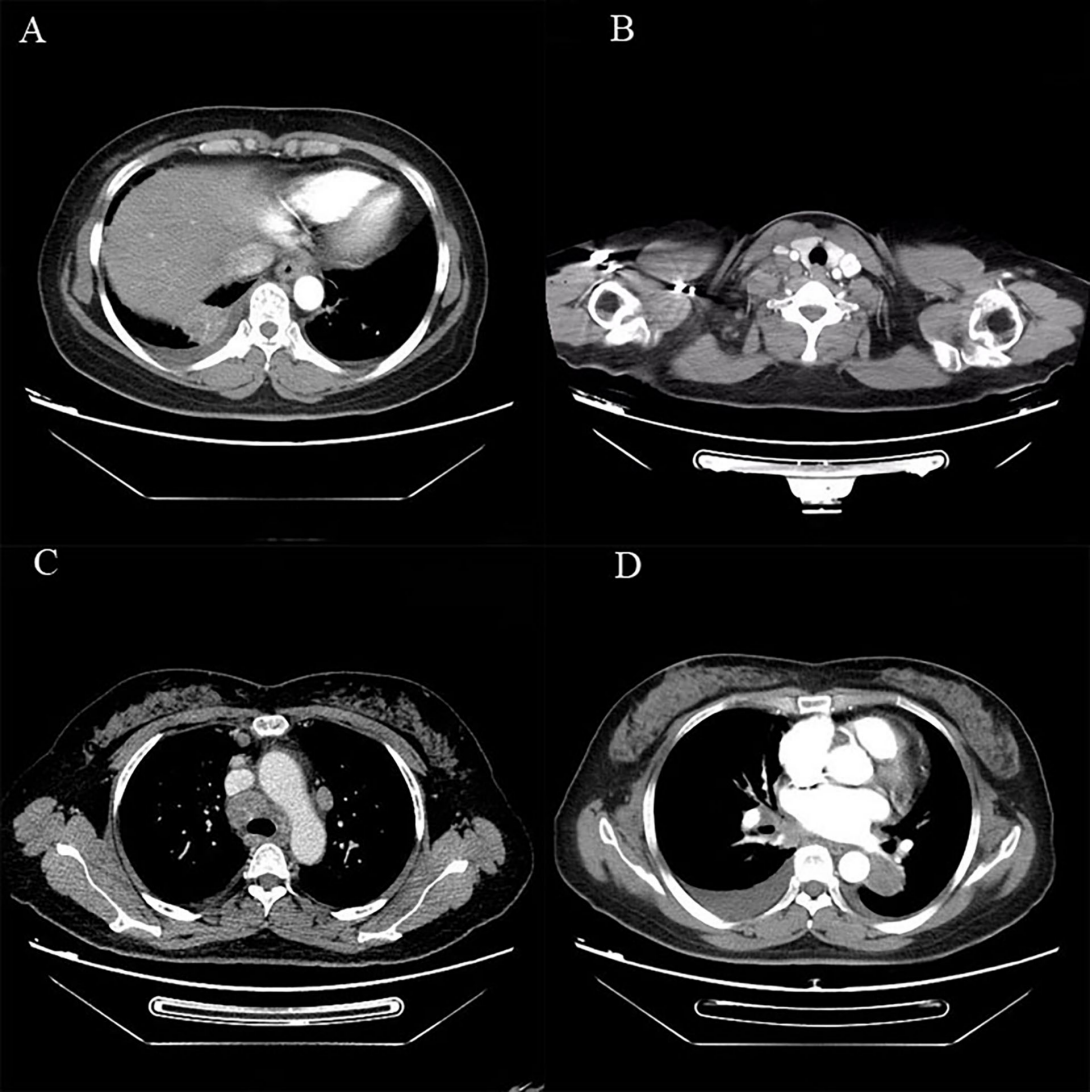
Chest CT of the patient before treatment: **(A–D)** show the range of lesions, respectively.

**Figure 2:**
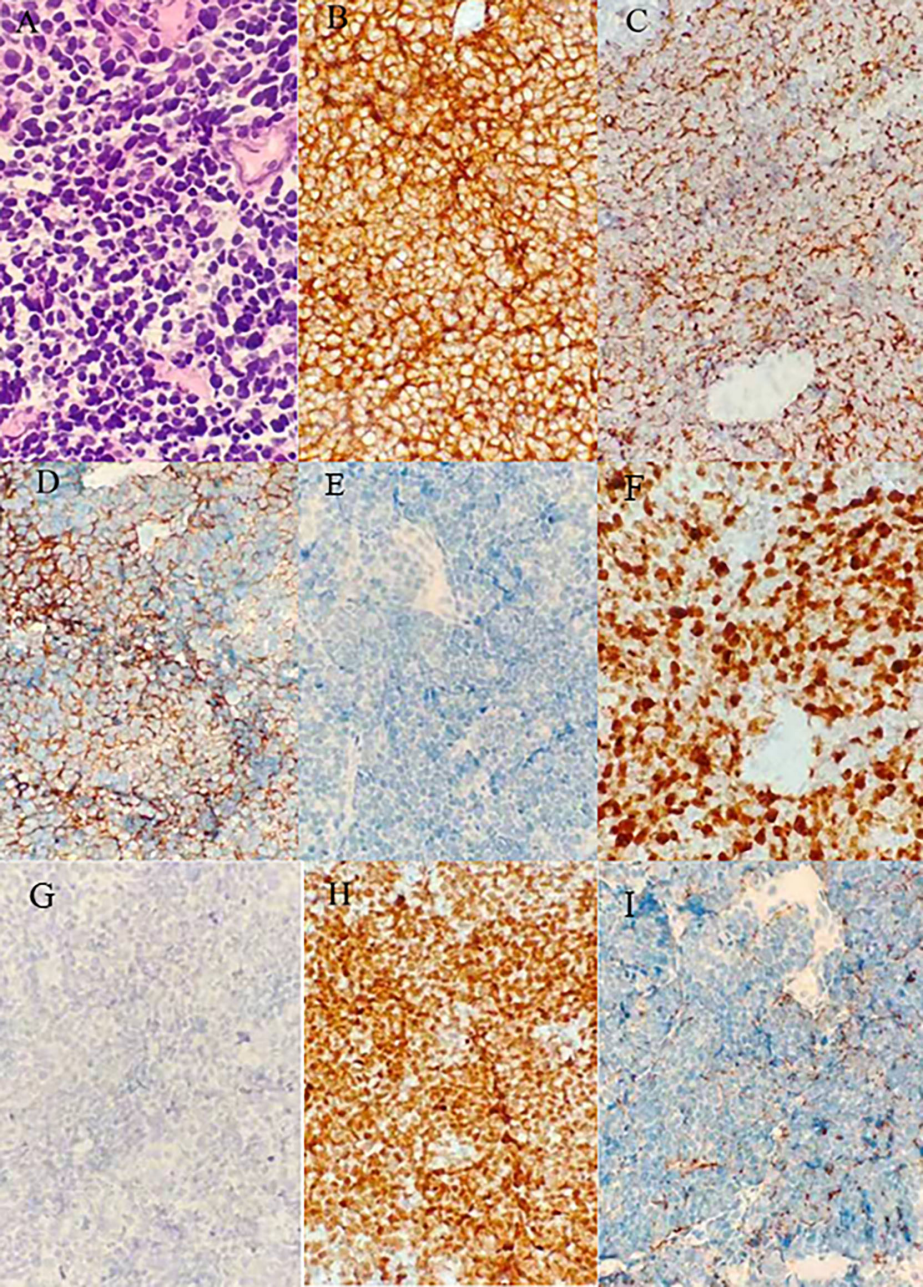
Results of the first puncture: **(A)** Microphotographs of small cell lung cancer; (**B–I**) thymic carcinoma with immunohistochemistry for CD56, CK, Syn, LCA, Ki67, P40, TTF-1, CgA.

**Figure 3:**
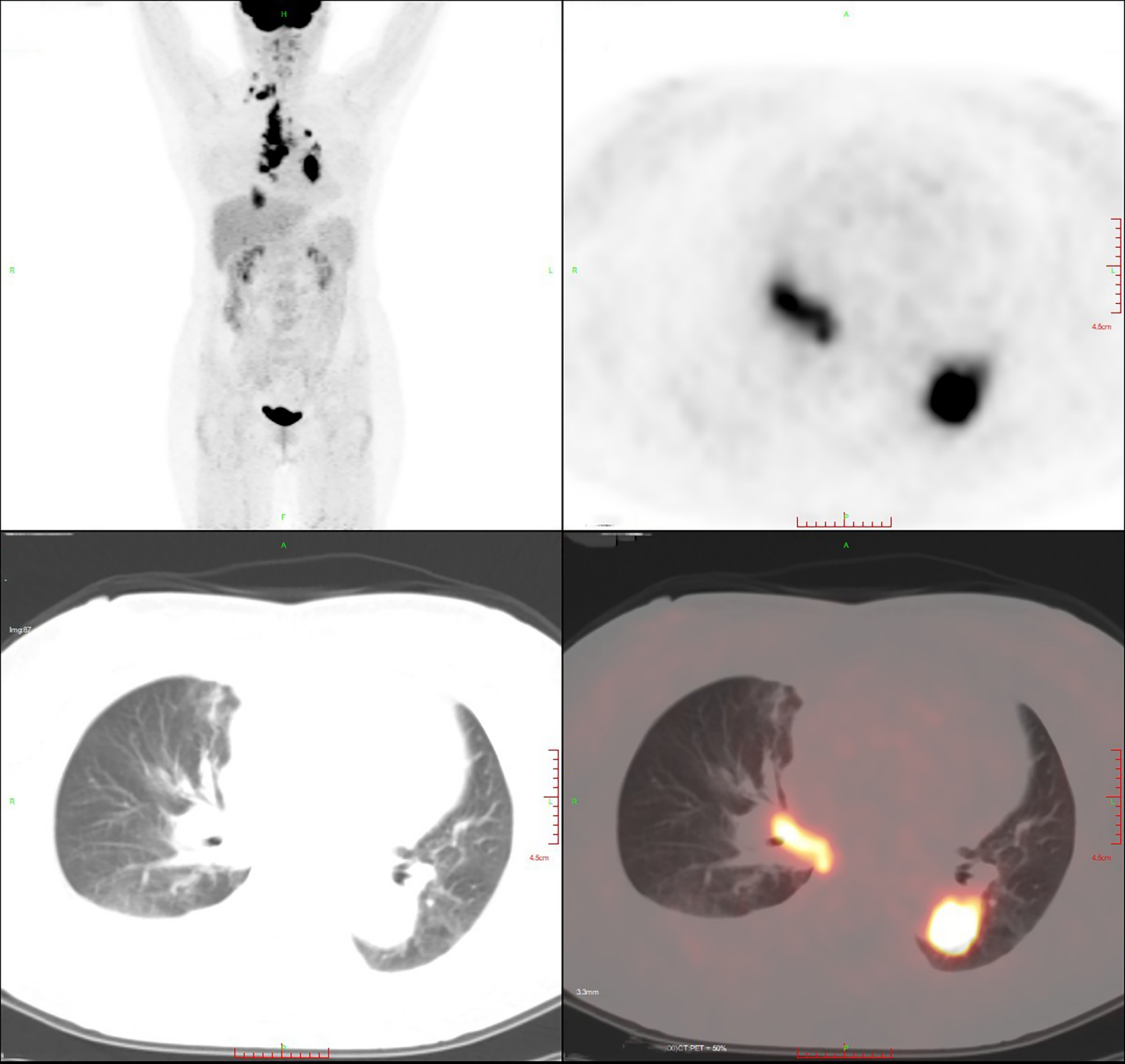
The lesions on positron emission tomography/computed tomography (PET/CT).

On April 8, 2019, a 56-year-old woman visited our hospital for chronic persistent cough. The patient’s symptoms lasted for 2 weeks without sputum or chest suffocation. Physical examination revealed an enlarged right-sided supraclavicular lymph, with a diameter of 2 cm, toughness, and an unclear boundary with the surrounding area. The respiratory sound of both lungs was normal. The patient had a family history of cancer, with his father dying of liver cancer and his mother suffering from bile duct cancer. Contrast-enhanced computed tomography (CT) of the chest showed mass in the lower lobe of the left lung and the right lower lung, which suggested that lung cancer might be considered (**Figures 1A–D**). On April 9, 2019, a lymph node biopsy was performed under ultrasound guidance, and the pathologic results showed small cell lung cancer (SCLC) (**Figures 2A–I**). Later, on April 15, 2019, positron emission tomography/computed tomography (PET/CT) showed positive lesions in the right lower lobe, mediastinal soft tissue, left lower lobe, bilateral hilum, and other sites (**Figure 3**).

**Figure 4:**
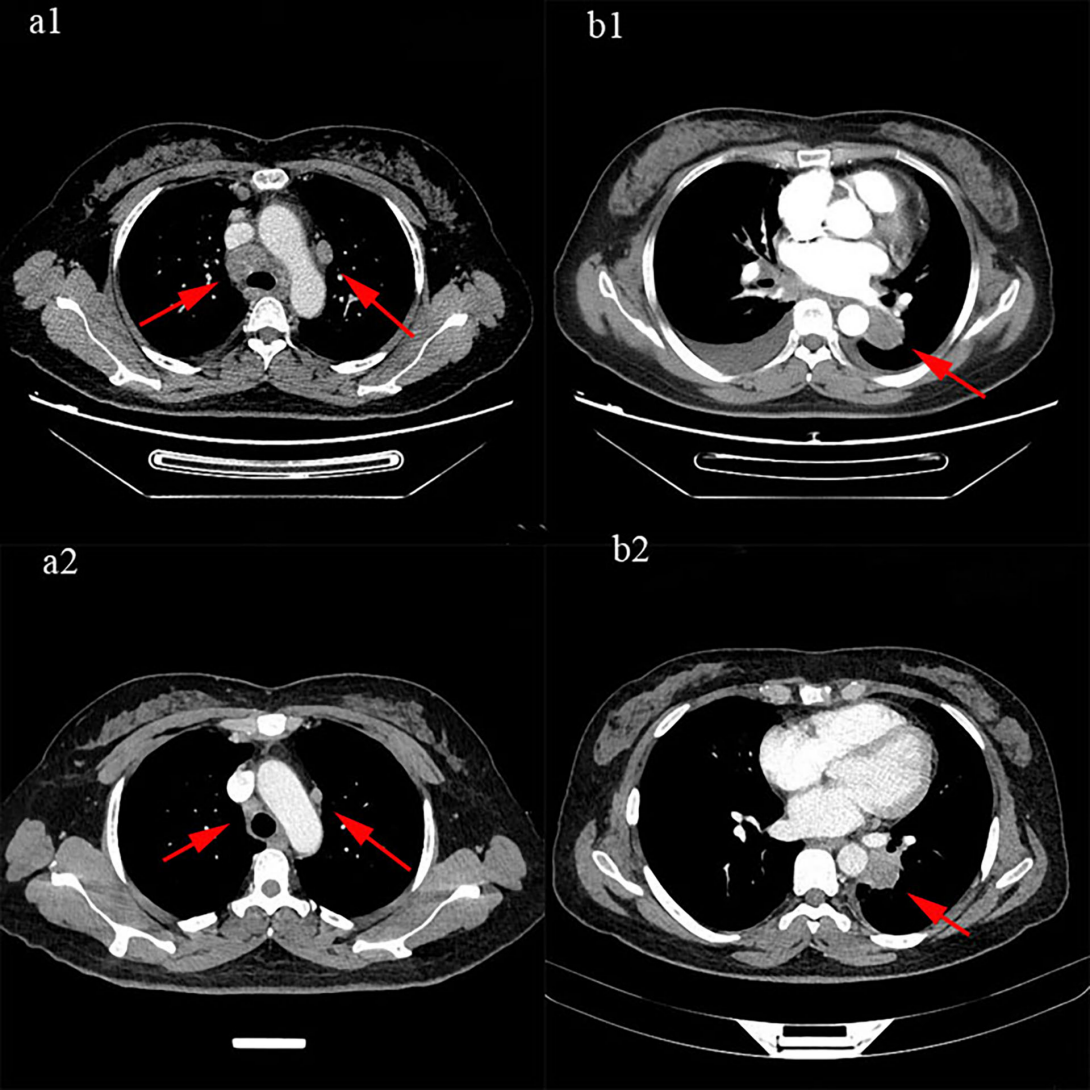
The efficacy was evaluated as partial response (PR): **(a1, b1)** were before treatment, **(a2, b2)** were after 2 cycles of treatment.

**Figure 5:**
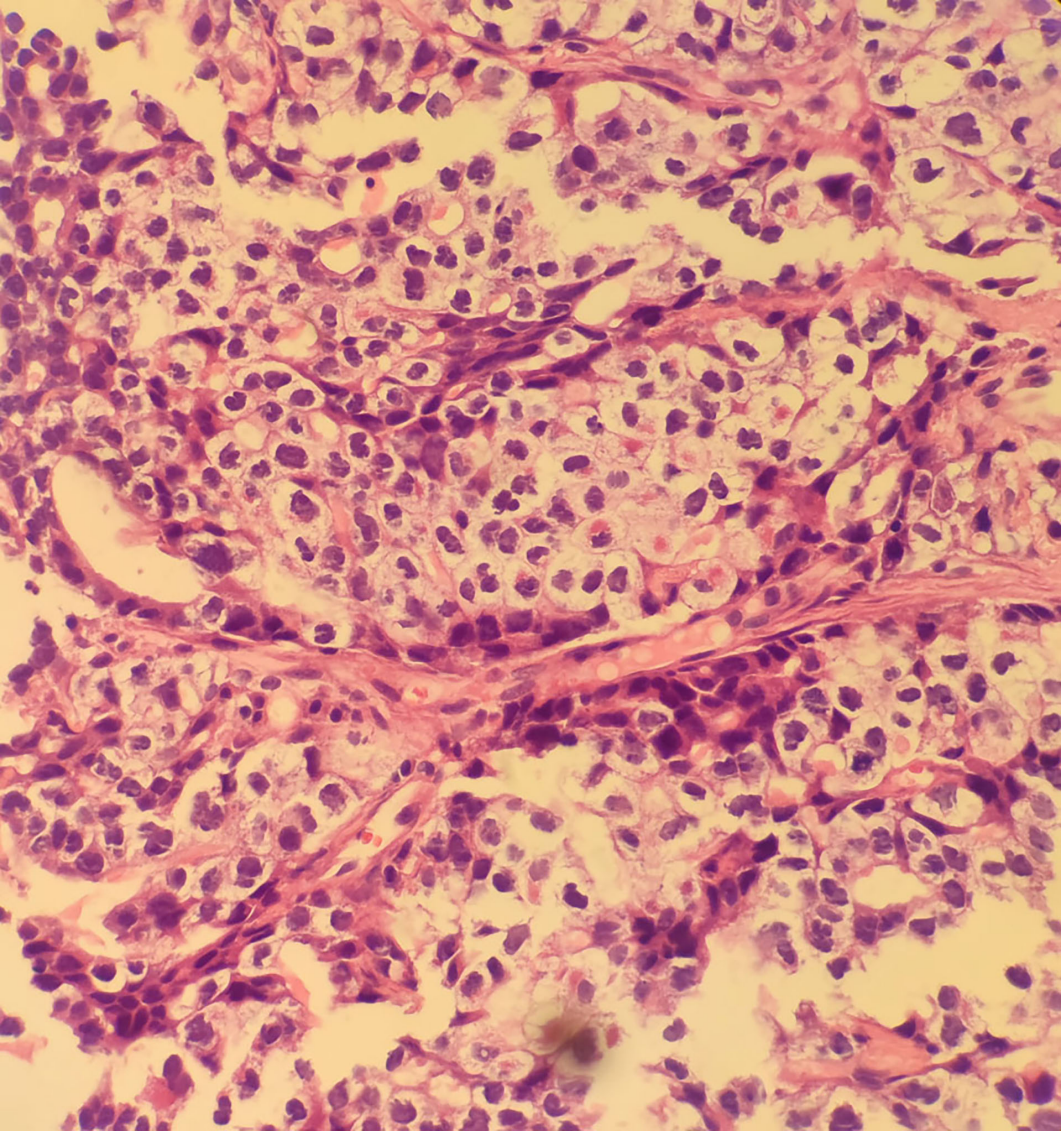
Result of the second puncture: lung adenocarcinoma.

**Figure 6:**
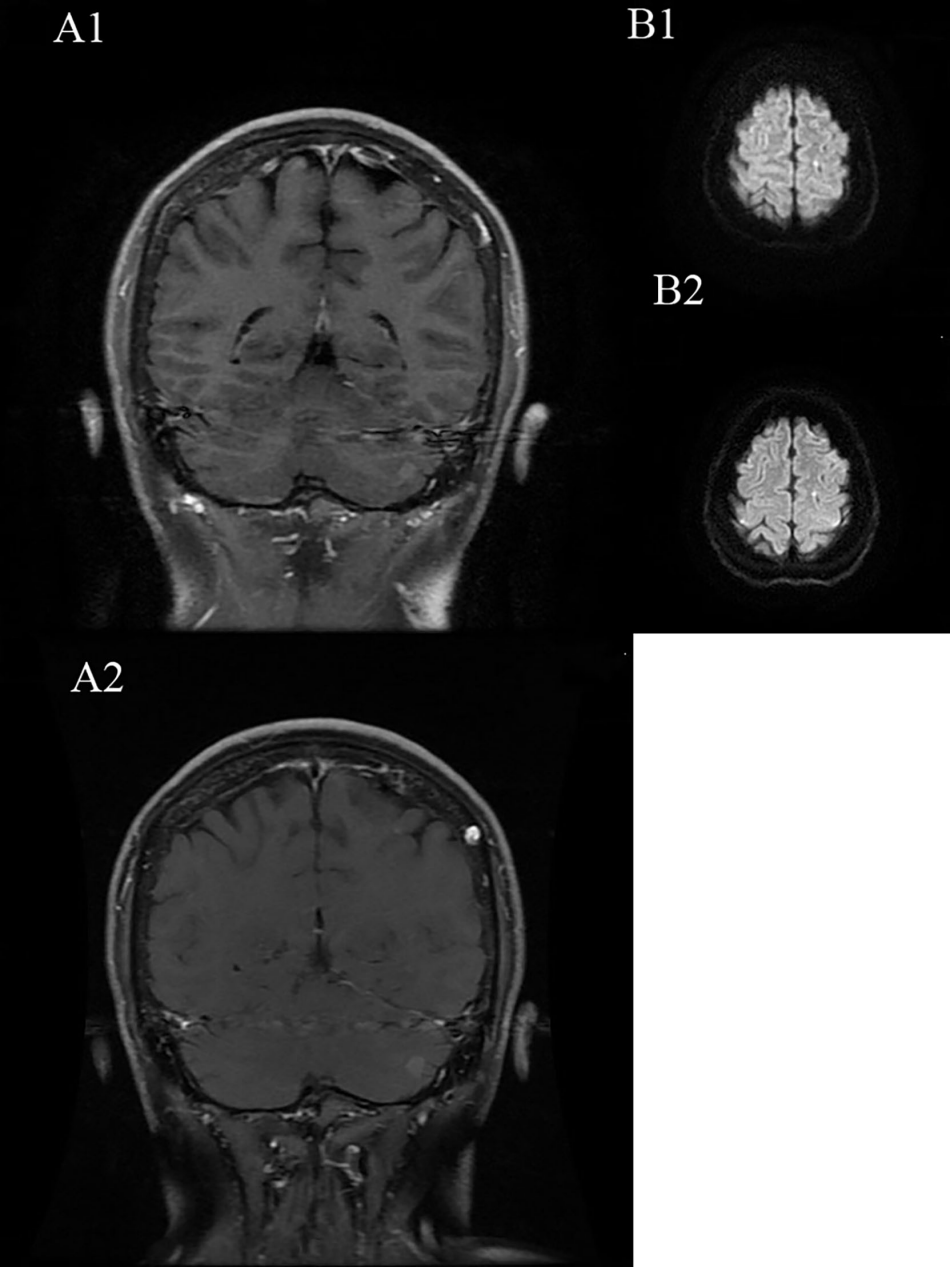
**(A1, B1)** are before treatment of alectinib, **(A2, B2)** are after treatment of alectinib.

According to American Joint Committee on Cancer (AJCC)/Union for International Cancer Control (UICC) 8th Tumor Node Metastasis (TNM) staging classification, the patient was initially diagnosed with extensive small cell lung carcinoma (ES-SCLC), which was defined as clinical stage T2aN3M1 and Siewert type IV. Moreover, according to the 2019 National Comprehensive Cancer Network (NCCN) guidelines for SCLC, chemotherapy is the first recommendation. Ruling out the taboo of chemotherapy from April 2019 to May 2019, the patient received 2 cycles of EP chemotherapy regimen with etoposide 100 mg/m^2^ on days 1–3 and cisplatin 25 mg/m^2^ on days 1–3 in each 3-week period. After 2 cycles of chemotherapy, the efficacy was evaluated as partial response (PR) (**Figure 4**). However, a chest CT taken on May 24, 2019, showed no significant change in the left mass, implying the difference in histological types between the left and right. Therefore, on May 27, 2019, a puncture biopsy of the left lung mass was performed. The pathology showed a low differentiated carcinoma, consistent with adenocarcinoma, with the possibility of complex carcinoma (**Figure 5**). Additional lung cancer common genetic testing with next-generation sequencing (NGS) indicated fusion of EML4 and ALK. Therefore, we modify the diagnosis of the patient with a terminal SCLC in the lower lobe of the right lung (cT2aN3M0, IIIB) and ALK mutation adenocarcinoma of the lower lobe of the left lung (cT4N2M0 IIIB, ALK mutation). According to the NCCN and Chinese Society of Clinical Oncology (CSCO) guidelines in 2019, we reformulated the treatment plan: 1) oral targeted drug treatment (crizotinib, 250 mg bid.); 2) continuous EP chemotherapy regimen; and 3) sequential radiotherapy (from July 19 to August 23, 2019). Sequential radiotherapy began after 4 cycles of chemotherapy. Moreover, the efficacy of the right lung (SCLC) after a 4-cycle chemotherapy was evaluated as PR. The patient received proton radiotherapy (4,500 cGy in total) for the tumors in the right lower lobe, mediastinum, and right supraclavicular lymph node area and then was treated by 6-MV X-ray arc intensity modulation (2,250 cGy) from July 19 to August 23, 2019. The fifth-cycle chemotherapy was completed on July 11, 2019. The efficacy of crizotinib and radiotherapy was evaluated. According to the abnormal signals in the left frontal lobe and cerebellar hemispheres shown by brain enhancement magnetic resonance (MR) (**Figures 6A1, B1**), the possibility of metastases was considered to be high. Just through imaging, we cannot judge where the brain metastases lesion came from, and both small cell lung cancer and lung adenocarcinoma with ALK-EML4 are possible. Fortunately, the patient has no symptoms of craniocerebral metastasis, and non-radiotherapy for intervention was considered. As we have known, the low concentration of cerebrospinal fluid is the common cause of crizotinib treatment failure. Moreover, some studies (7, 8) have shown that alectinib has a better effect on craniocerebral metastasis than crizotinib, so we chose alectinib. A month later, we rechecked the brain enhancement MR (**Figures 6A2, B2**) and found that the lesions did not shrink and there were new lesions. Therefore, we considered that the brain’s metastases might be from SCLC. Therefore, the patient underwent a 6-MV X-ray whole-brain arc intense-modulated radiotherapy; the metastatic tumor increased starting from July 19, 2019, to August 23, 2019. At the subsequent 2-year follow-up after completion of radiotherapy, this patient was without evidence of locally recurrent or distant disease.

## Discussion

Clinically, MPLCs are commonly seen as adenocarcinoma, which often need to be identified with intrapulmonary metastasis (IM) of lung cancer, and the treatment methods of the two are completely different. Studies found that there was a significant difference in prognosis between MPLC and IM. Therefore, it is very important to identify MPLCs, especially sMPLC and IM. In differentiating MPLCs from IM, we can use histopathology, imaging, and molecular genetics. In general, MPLCs have different histological types and have different *in situ* carcinogens, while IM has the same histological type and the same origin. In addition, we can also distinguish between the two based on imaging results (9, 10). Currently, most of the MPLCs reported are mainly multifocal adenocarcinoma, and there are few cases like the one in this case, which is also the unique feature of this case.

In the diagnosis of MPLCs, we still need to distinguish it from combined small cell lung cancer (c-SCLC). C-SCLC is defined by the World Health Organization (WHO) as SCLC combined with additional components that consist of any of the histological types of NSCLC, such as usually adenocarcinoma (ADC), squamous-cell carcinoma (SCC), large-cell carcinoma (LCC), large-cell neuroendocrine carcinoma (LCNEC), or less commonly spindle-cell carcinoma or giant-cell carcinoma (11–15). C-SCLC generally occurs in the same lobe and is mostly interrelated, while multiple primary lung cancers are more likely to occur in different lobes or in different lungs. In this case, two lesions were located in two different lung lobes. During the treatment, different therapeutic effects suggest that the pathological sources of two lesions may be different, and the second biopsy confirmed our hypothesis.

**Figure 7:**
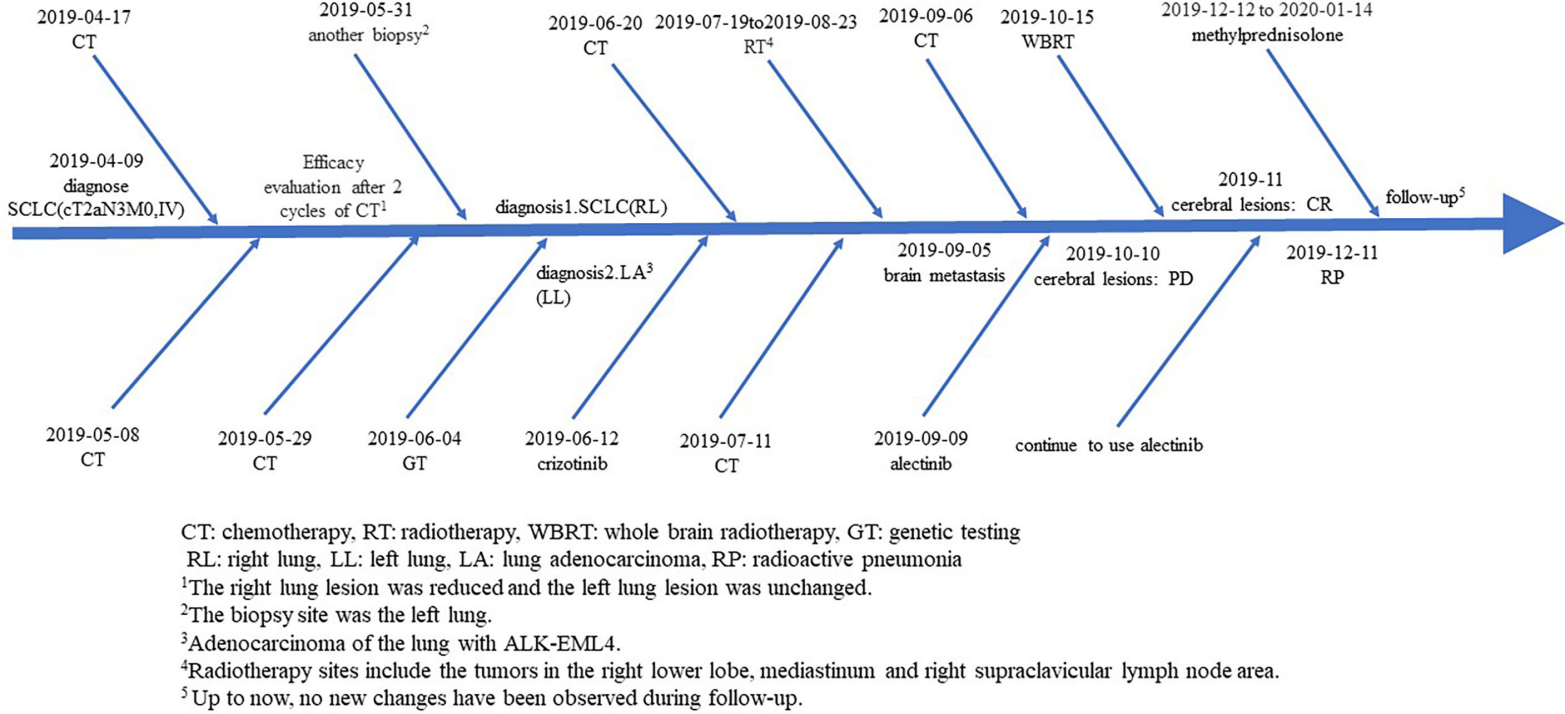
Flow chart of patient treatment.

At present, there is still no unified understanding of the treatment of MPLCs. However, the treatment principles of MPLCs in domestic and foreign literature are consistent, and they all believe that as long as there is no absolute contraindication, active local treatment based on surgery should be carried out, combined with the multidisciplinary comprehensive treatment mode of adjuvant radiotherapy and chemotherapy (16–18). Advanced MPLC patients can be treated with radiotherapy, chemotherapy, targeted therapy, interventional therapy, immunotherapy, best support therapy, and other palliative treatments. In this case, multiple mediastinal and supraclavicular lymph node metastases suggest that the disease is at least locally advanced, regardless of the pathological type, so surgery was not one of the first choices. Referring to the 2019 NCCN guidelines and CSCO guidelines, combined with the specific condition of the patient, we adopted chemotherapy for SCLC, TKI-targeted drugs for ALK rearrangement adenocarcinoma, and sequential radiotherapy for right lung lobe lesion, mediastinum, supraclavicular lymph node, and brain (**Figure 7**). Furthermore, in the whole course of treatment, the patient also had some treatment-related adverse reactions, such as grade 1 nausea and vomiting, grade 1 bone marrow suppression, and grade 2 radiation pneumonia. Methylprednisolone was used to treat radioactive pneumonia. Through symptomatic treatment, the patient achieved a good quality of life.

Compared to other cases of MPLCs, our case has a unique feature. In this case, the origin of the brain metastasis (BM) is a matter of consideration, because it determines the treatment plan. Some studies (11, 19, 20) showed that the incidence of BM in NSCLC patients is 30%–40%, and lung cancer is responsible for approximately 50% of all BM. However, the molecular mechanism of BM in lung cancer is still unclear and may be related to the interaction of the blood–brain barrier, cancer stem cells, lung cancer cells, and brain microenvironment (21). Brain radiotherapy is the best treatment for SCLC patients with brain metastasis, and small molecule targeted drugs are another choice for NSCLC patients with gene mutation (11, 22–24). In our case, we cannot estimate where the brain metastases came from just according to MR imaging. Moreover, the patient has no symptoms of craniocerebral metastasis; therefore, we have time to choose another TKI drug, alectinib. Several studies have shown that alectinib has a better effect on craniocerebral metastasis than crizotinib. It is a pity that after a 1-month treatment of alectinib, the craniocerebral lesions did not shrink and new lesions appeared. At this time, we highly suspected that the craniocerebral lesions might be from SCLC. Then, brain radiation was given to the patient and the BM was reduced. Diagnosis and treatment complement each other. Only when the diagnosis is clear can the right medicine be applied.

## Conclusion

In conclusion, diagnosis is the first priority for the treatment of diseases. With the discovery of driver mutations in lung adenocarcinoma (ADC), next-generation sequencing (NGS) would provide an explicit answer to the key question, whether individual tumors represent intrapulmonary metastases or independent tumors. Only when the diagnosis is correct can we choose the right treatment method and benefit patients more. Moreover, whether the disease is rare or common, patients benefit from systematic treatment. For some rare diseases, we should start with diagnosis, progress step by step, and overcome them one by one. Further study is warranted for the diagnosis and treatment of MPLCs.

## Data Availability Statement

The original contributions presented in the study are included in the article/supplementary material. Further inquiries can be directed to the corresponding author.

## Ethics Statement

The studies involving human participants were reviewed and approved by the Ethics Committee of Affiliated Hospital of Qingdao University Affiliated Hospital of Qingdao University. The patients/participants provided their written informed consent to participate in this study. Written informed consent was obtained from the individual(s) for the publication of any potentially identifiable images or data included in this article.

## Author Contributions

WJ, YZ, and FL contributed to the conception and design of the study. QQ and YX organized the database. ZH and WX wrote the first draft of the manuscript. All authors contributed to the manuscript revision and read and approved the submitted version.

## Conflict of Interest

The authors declare that the research was conducted in the absence of any commercial or financial relationships that could be construed as a potential conflict of interest.

## Publisher’s Note

All claims expressed in this article are solely those of the authors and do not necessarily represent those of their affiliated organizations, or those of the publisher, the editors and the reviewers. Any product that may be evaluated in this article, or claim that may be made by its manufacturer, is not guaranteed or endorsed by the publisher.
